# Disturbances in affective touch in hereditary sensory & autonomic neuropathy type III

**DOI:** 10.1016/j.ijpsycho.2014.04.002

**Published:** 2014-07

**Authors:** Vaughan G. Macefield, Lucy Norcliffe-Kaufmann, Line Löken, Felicia B. Axelrod, Horacio Kaufmann

**Affiliations:** a School of Medicine, University of Western Sydney, Australia; b Neuroscience Research Australia, Sydney, Australia; c Dysautonomia Center, NYU Langone Medical Center, NY, USA; d Oxford Centre for Functional MRI of the Brain (FMRIB), University of Oxford, John Radcliffe Hospital, Oxford OX3 9DU, UK

**Keywords:** Affective touch, CT afferents, Pleasant touch, Tactile sensation

## Abstract

Hereditary sensory and autonomic neuropathy type III (HSAN III, Riley–Day syndrome, Familial Dysautomia) is characterised by elevated thermal thresholds and an indifference to pain. Using microelectrode recordings we recently showed that these patients possess no functional stretch-sensitive mechanoreceptors in their muscles (muscle spindles), a feature that may explain their lack of stretch reflexes and ataxic gait, yet patients have apparently normal low-threshold cutaneous mechanoreceptors. The density of C-fibres in the skin is markedly reduced in patients with HSAN III, but it is not known whether the C-tactile afferents, a distinct type of low-threshold C fibre present in hairy skin that is sensitive to gentle stroking and has been implicated in the coding of pleasant touch are specifically affected in HSAN III patients. We addressed the relationship between C-tactile afferent function and pleasant touch perception in 15 patients with HSAN III and 15 age-matched control subjects. A soft make-up brush was used to apply stroking stimuli to the forearm and lateral aspect of the leg at five velocities: 0.3, 1, 3, 10 and 30 cm/s. As demonstrated previously, the control subjects rated the slowest and highest velocities as less pleasant than those applied at 1–10 cm/s, which fits with the optimal velocities for exciting C-tactile afferents. Conversely, for the patients, ratings of pleasantness did not fit the profile for C-tactile afferents. Patients either rated the higher velocities as more pleasant than the slow velocities, with the slowest velocities being rated unpleasant, or rated all velocities equally pleasant. We interpret this to reflect absent or reduced C-tactile afferent density in the skin of patients with HSAN III, who are likely using tactile cues (i.e. myelinated afferents) to rate pleasantness of stroking or are attributing pleasantness to this type of stimulus irrespective of velocity.

## Highlights

• C-tactile afferents in hairy skin are believed to mediate affective touch.
• They are sensitive to slow brushing stimuli, which are perceived as pleasant.
• It is not known whether C-tactile afferents are affected in HSAN III.
• Ratings of pleasantness were reduced in 15 HSAN III patients compared to controls.
• We suggest that the density of C-tactile afferents is reduced in HSAN III.

## 1 Introduction

The hereditary sensory and autonomic neuropathies (HSAN) are a group of rare neurological disorders with sensory and, to a varying degree, autonomic deficits that can be classified into five types, depending on mode of inheritance, neuropathology and clinical symptoms (Dyck et al., 1983). HSAN III—also known as Riley–Day syndrome or, more commonly, familial dysautonomia—is a rare autosomal recessive disorder that is largely limited to individuals of Eastern European Jewish descent. It has been attributed to a single point mutation on chromosome 9q (Blumenfeld et al., 1993), affecting the production of IKAP/ELP1 protein (Slaugenhaupt et al., 2001). Depletion of this protein results in reduced transcriptional elongation of target genes required for cell motility, suggesting that defective cellular motility may underlie much of the developmental neuropathology of HSAN III (Close et al., 2006; Cohen-Kupiec et al., 2011). The phenotype becomes apparent in infancy, with difficulties in swallowing and frequent lung infections, hypotonia, an inability to generate tears and an absent flare response to intradermal histamine being key indicators (Riley et al., 1949; Axelrod, 2002). In childhood and adolescence affected individuals present with very labile blood pressures, with orthostatic hypotension and greatly elevated blood pressures during episodes of anxiety; these may precipitate into autonomic crises and vomiting (Riley et al., 1949, Norcliffe-Kaufmann et al., 2010, 2013; Macefield et al., 2013a). They also exhibit elevated temperature thresholds (Hilz and Axelrod, 2000; Hilz et al., 2004), an indifference to pain (Riley et al., 1949; Dyck et al., 1983; Axelrod, 2002; Hilz et al., 2004), and absent tendon and H-reflexes (Aguayo et al., 1971; Macefield et al., 2011).

Using invasive microelectrode recordings from the peripheral nerves we recently showed that these patients have absent muscle spindle afferents, which we postulated may account for the absence of stretch reflexes, as well as their ataxic gait and disturbed proprioception (Macefield et al., 2011, 2013b). Interestingly, despite the absence of large-diameter muscle afferents in the same study we noted that large-diameter tactile afferents could be recorded from cutaneous fascicles (Macefield et al., 2011). Furthermore, the presence of histologically normal, myelinated, low-threshold mechanoreceptors in the skin (Winkelmann et al., 1966; Pearson et al., 1975) in patients with HSAN III would suggest that tactile sensibility is normal, though it is curious that affected individuals do appear to have a heightened sensitivity to tactile stimulation; indeed, the patients have been described as being abnormally ticklish (Mahloudji et al., 1970). Nevertheless, patients with HSAN III have a greatly reduced number of unmyelinated axons in the skin (Aguayo et al., 1971; Pearson et al., 1975; Dyck et al., 1978; Hilz et al., 2004), which accounts for their greatly elevated thresholds to hot and cold stimuli applied to the skin (Hilz and Axelrod, 2000; Hilz et al., 2004).

The purpose of the present study was to examine a specific element of tactile sensibility in HSAN III: the capacity to perceive gentle brushing of the skin as being pleasant. This aspect of touch, referred to as *affective touch*, is believed to be subserved by a specific class of unmyelinated afferent with low-mechanical thresholds (Vallbo et al., 1999; McGlone et al., 2007). Termed C-tactile (CT) afferents, these slowly-conducting afferents are found only in hairy skin and have not been detected in the glabrous skin of the palms (Olausson et al., 2010). The afferents are very sensitive to innocuous tactile stimulation (Nordin, 1990; Vallbo et al., 1999; Wessberg et al., 2003), preferentially responding to gentle stroking over the skin at 1–10 cm/s; compared to slower and faster brushing velocities this range is also rated as being most pleasant (Essick et al., 1999; Löken et al., 2009), with ratings of pleasantness exhibiting an inverted U profile as a function of brushing velocity (Löken et al., 2009). In the current study we used the same experimental paradigm–brushing the skin at velocities of 0.3–30 cm/s–employed by Löken et al. (2009) to test the hypothesis that affective touch is compromised in patients with HSAN III. Moreover, while Löken et al. only examined affective touch by brushing the forearm, in the current study we also assessed the function of C-tactile afferents in the leg—which are also known to contain C-tactile afferents (Löken et al., 2007)—because it is known that sensory disturbances in the lower limbs are greater than in the upper limbs in HSAN III (Macefield et al., 2011). In addition to assessing C-tactile function in HSAN III, we also examined thermal and vibration thresholds.

## 2 Methods

Experiments were performed on 15 patients (10 female, 5 male; 14–48 years, mean ± SE 27.0 ± 2.8 years) with hereditary sensory and autonomic neuropathy type III (HSAN III); molecular confirmation of diagnosis was available for all patients. All patients were recruited from the database of the Dysautonomia Center at New York University Medical Center and gave informed consent to the procedures, which were approved by the Institutional Review Board of the New York University Medical Center. All studies were performed in accordance with the Declaration of Helsinki. Data from one of the HSAN III patients, a 15 year-old female, and control data from 15 age-matched healthy subjects (11 female, 4 male; 14–47 years, 27.3 ± 2.6 years), were obtained in the corresponding author's laboratory in Sydney. Participants were seated at a table in a comfortable chair with the left leg supported in the extended position. Stroking was performed with a 4 cm diameter sable makeup brush (Cheek Brush #6, Lancôme, NY, USA) over a 10 cm strip of skin: (i) on the anterolateral aspect of the left forearm, in the area innervated by the anterior brachiocutaneous nerve and (ii) on the lateral aspect of the left leg, in the area distal to the knee innervated by the lateral cutaneous fascicle of the common peroneal nerve. Single brush stimuli were applied by one investigator (VM) in the distal–proximal direction at one of five velocities: 0.3, 1, 3, 10 and 30 cm/s. A graphical display on a computer monitor provided the investigator with a vertical line that moved at the set velocity. Six trials were conducted at both the forearm and leg sites, each trial comprising a single brush stroke at each of the five velocities, each velocity presented in a quasi-random order. The experimenter did not speak to the subject during or between trials. Subjects were provided with a sheet of paper on which five 10 cm horizontal lines were provided, each corresponding to a single brushing trial. The lines included a short vertical line indicating the mid point (5 cm), with the anchor word “unpleasant” and emoticon (sad face) provided at the far left (0 cm) and “pleasant” (and a happy face emoticon) at the far right (10 cm). Subjects were provided with a pen to mark with a vertical line their rating of pleasantness following the single brush stroke. There was no time limit imposed for the subject to complete his or her rating, which was typically completed within 10 s, following which another brush stroke was applied. The point on the horizontal line intersected by the subject's mark was measured and the length of the line was used as the subjective rating. In each subject mean rating values were computed from all six trials. Hot and cold thermal thresholds were assessed using Peltier-element thermodes placed over the left thenar eminence; the baseline (reference) temperature was 32 °C. (TSA II Neurosensory Analyser, Medoc, Israel). Vibration perception thresholds were measured from the metacarpophalangeal and metatarsoplangeal joints of the thumb and hallux of both limbs at 120 Hz (Bio-thesiometer; BioMedical Instrument Company, Newbury, OH, USA). Statistical analysis was performed using 2-way ANOVA and multiple *t*-tests with Holm–Sidak correction for multiple comparisons, or non-paired *t*-tests for group differences (Prism 6 for Mac OS X, Statsoft Inc, USA).

## 3 Results

**Fig. 1:**
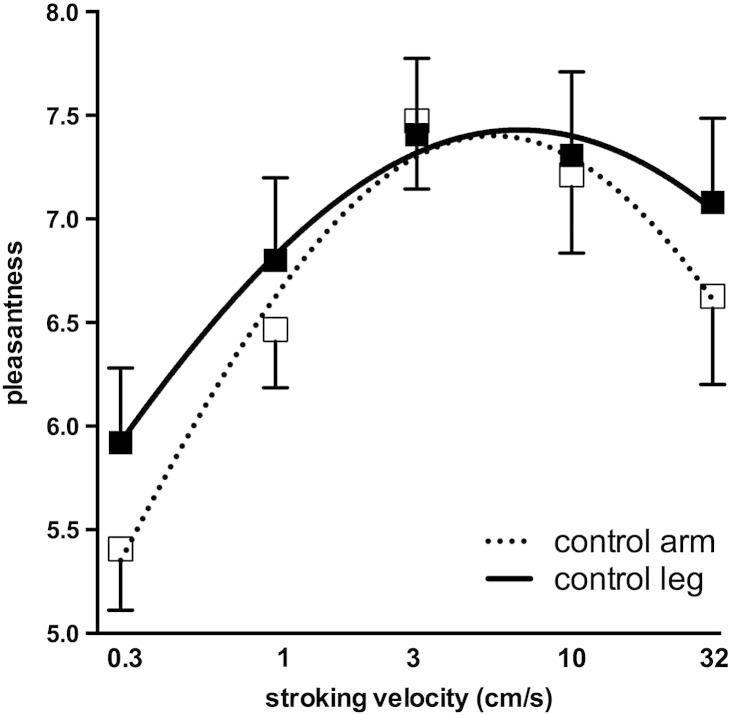
Rating of pleasantness for brushing velocities of 0.3–30 cm/s applied to the forearm (*n* = 15) and lateral aspect of the leg (*n* = 12). The data have been fitted to a logarithmic Gaussian distribution. Brushing velocity is displayed logarithmically (base 2).

Fifteen patients with Hereditary Sensory and Autonomic Neuropathy type III (HSAN III) were studied and compared with 15 age-matched control subjects. In controls, brush stroking on the forearm generated ratings of pleasantness that were consistent with previously published results from two other groups of healthy subjects (Löken et al., 2009; Morrison et al., 2011): specifically, subjects rated the 3 cm/s velocity as being the most pleasant, with the slowest velocity (0.3 cm/s) being considered the least pleasant. The highest velocity (30 cm/s) was considered less pleasant than the 3 cm/s stimulus. Applying the same brushing stimuli to the lateral aspect of the leg produced ratings that were not statistically different from those obtained for the forearm (F_[1, 140]_ = 1.300, *P* = 0.2561), as assessed by two-way analysis of variance. It can be seen in Fig. 1 that the ratings for both the arm and leg exhibit an inverted U profile; these were fitted to a logarithmic Gaussian distributions.

**Fig. 2:**
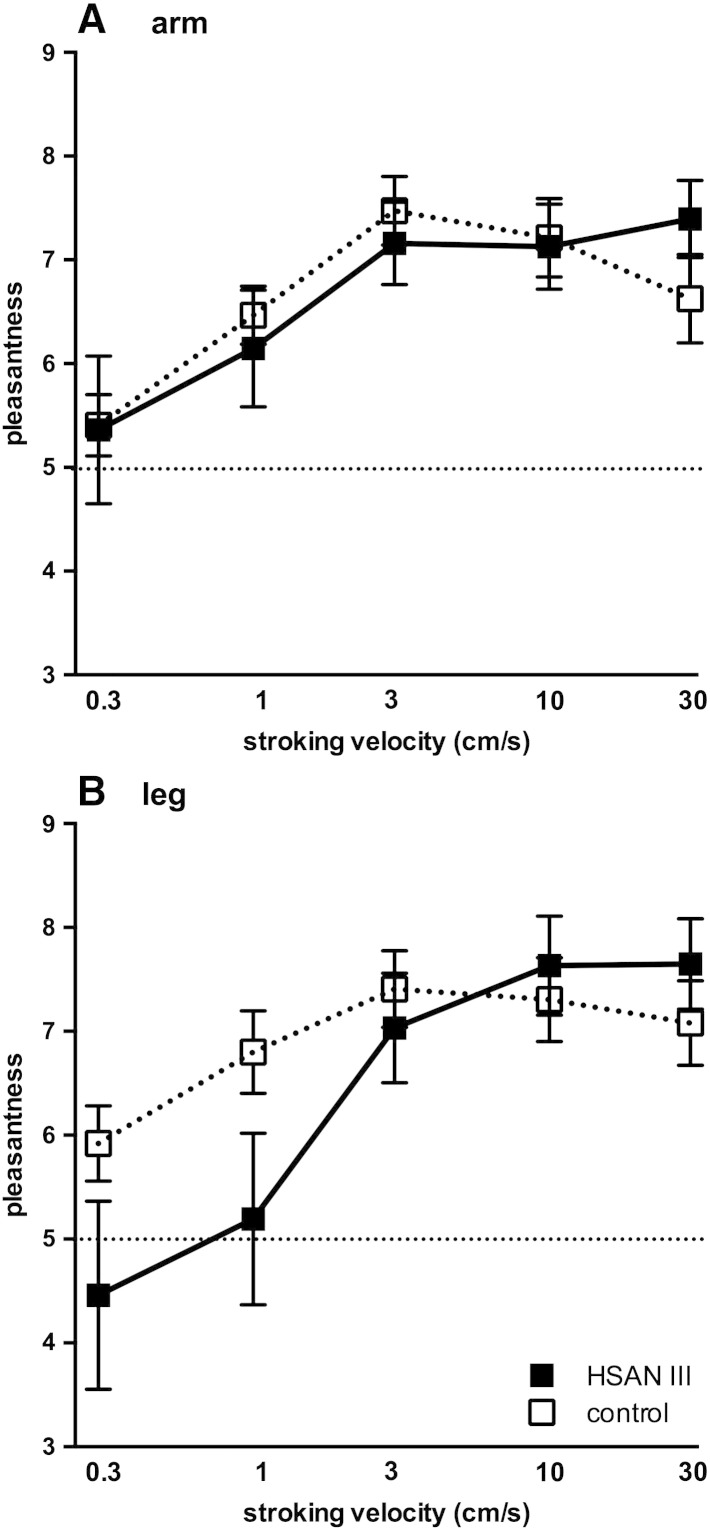
Rating of pleasantness for brushing velocities of 0.3–30 cm/s applied to the forearm (**A**) and lateral aspect of the leg (**B**). Number of participants = 15 for both the HSAN III and control subjects, with the exception of the leg (*n* = 12 for HSAN III patients, *n* = 15 for control subjects). Scores above 5 (dashed horizontal line) are considered pleasant; scores below 5 are considered unpleasant. Stroking velocity is displayed logarithmically (base 2).

Fig. 2A shows the mean ratings of pleasantness following forearm brushing for all 15 control subjects (broken lines) and 15 HSAN patients (solid lines). Two-way ANOVA showed that there was a clear effect of brushing velocity on ratings (F_[4, 140]_ = 6.790, *P* < 0.0001) but no significant differences between the two groups (F = 0.0, *P* > 0.99). Data for the leg are shown in Fig. 2B; because of modesty issues three patients with HSAN did not want to have their legs stroked, so data are only available for 12 subjects. As for the arm, the velocity of brushing applied to the leg had a significant effect on ratings (F_[4, 125]_ = 7.554, *P* < 0.0001), but ratings of pleasantness were significantly lower in the patients than the controls at the slowest brushing velocities (0.3 and 1 cm/s; *p* < 0.05, *t*-tests corrected for multiple comparisons). Two-way ANOVA revealed no significant difference in ratings between the arm and leg for the patients (F_[1, 125]_ = 0.4417, *P* = 0.5075).

**Fig. 3:**
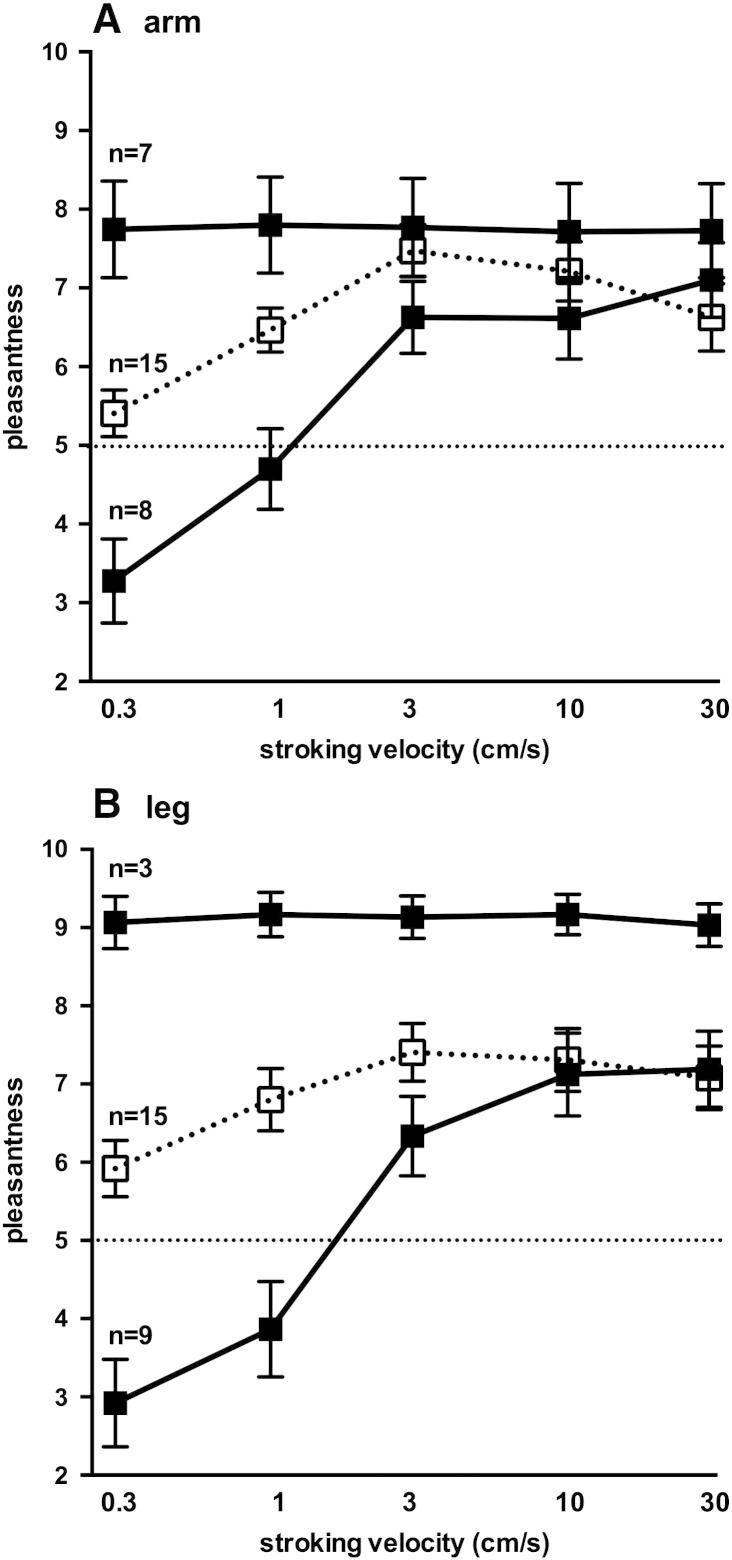
Rating of pleasantness for brushing velocities of 0.3–30 cm/s applied to the forearm (**A**) and lateral aspect of the leg (**B**). For both the arm and the leg, the data for the HSAN III patients have been separated into two profiles: a flat response to changes in brushing velocity and one that was affected by brushing velocity; *n* values refer to number of patients comprising each group. Data for the 15 control subjects are shown for comparison. Scores above 5 (dashed horizontal line) are considered pleasant; scores below 5 are considered unpleasant. Stroking velocity is displayed logarithmically (base 2).

It is apparent that variability in rating was very high at these low speeds for the patients, as shown by the large standard deviation for both the arm (Fig. 2A) and leg (Fig. 2B); a post-hoc analysis of the individual data provides an explanation. As seen in Fig. 3, there were two distinct response patterns to brushing that allowed us to divide patients into two groups: one in which the brushing stimuli were rated as being equally pleasant at all velocities, and another group in whom the slowest velocities were rated as unpleasant and the higher velocities as pleasant. There were no differences in mean age between the two groups (28 ± 4 vs 26 ± 4 years) and no differences in gender (there were 5 females in both groups) that could account for the two response patterns. Moreover, these patterns were similar for both the arm (Fig. 3A) and leg (Fig. 3 B), and—for the non-flat responses—both could be fitted to a semi-logarithmic linear regression (*r*^2^ = 0.47 for the arm and 0.51 for the leg). For the arm, ratings were significantly lower than the controls in a subgroup of eight patients at brushing velocities of 0.3 and 1 cm/s (*p* < 0.005, *t*-tests corrected for multiple comparisons); the same was true for the leg (*p* < 0.0001). While only three patients exhibited a flat response profile for the leg (and these also showed a flat profile for the forearm), it should be noted that data could not be obtained from three of the seven patients who exhibited a flat profile for the forearm; it is likely that these patients exhibited a flat profile for the leg as well.

Thermal and vibration perception thresholds for the patients are shown in Table 1. While hot and cold thresholds, as well as vibration thresholds, were elevated—as described by our laboratory previously (Hilz and Axelrod, 2000)—there were no significant differences in any parameter that could account for the differences in the stroking rating profile between the two groups.

## 4 Discussion

The aim of this study was to assess whether the function of C-tactile afferents was affected in patients with HSAN III. While pooling the data from all patients would suggest that there was no disturbance of function in the forearm, whereas there was in the leg, closer analysis of the data showed two distinct patterns of responses, neither of which fit the pattern expected for intact C-tactile afferent function. Some patients rated the brushing stimuli as being equally pleasant at all velocities, and it is likely that these individuals were attributing a positive hedonic value to these stimuli that was independent of stroke velocity, such as the “softness” of the brush itself. For the others, there was a quasi-sigmoidal relationship between brushing velocity and their rating; because the “inverted U” profile seen in the control subjects was absent in these patients it would appear that they were rating the pleasantness of the stimulus as a function of the firing rates of large-myelinated tactile afferents, which are known to increase their rate of discharge with brushing velocity (Löken et al., 2009). However, the plateau in the ratings at velocities of 3–30 cm/s does not fit with the firing rates of myelinated afferents in human hairy skin (slowly adapting type I, slowly adapting type II, field and hair units), which show a monotonic increase with increasing brushing velocity (Löken et al., 2009).

While mammals have Aδ axons that respond to fine hair movements (down hair units) (Burgess et al., 1968; Perl, 1968), C-tactile afferents are the only class of unmyelinated afferent that respond to light touch in the human forearm skin (Vallbo et al., 1995, 1999; Wessberg et al., 2003), and there is no reason to think that they are not present in the hairy skin of the leg. Microelectrode recordings from single tactile afferents have shown that brushing velocity affects C-tactile afferent discharge: velocities of 1–10 cm/s activate unmyelinated tactile C afferents more effectively than slower (0.1 and 0.3 cm/s) or faster (30 cm/s) velocities. Intermediate velocities are also perceived as more pleasant than slower or faster velocities (Löken et al., 2009). Accordingly, C-tactile afferents encode aspects of gentle stroking, perceived as being pleasant. However, affective perception is a construct of the combination of signal input from this affective touch system as well as the discriminative touch system. Indeed, it is known that in patients with large-fibre sensory neuropathy, in which discriminative touch is lost but C-tactile function is preserved, perception of pleasantness can only be reported using forced-choice methods, suggesting that the C-tactile system alone is less accessible to conscious reports and that large-fibre input also contributes to rating a tactile stimulus as pleasant (Olausson et al., 2002; Löken et al., 2011).

Recently, it was reported that the ability to sense affective touch in patients with HSAN V (Morrison et al., 2011) is markedly reduced. This consanguinous cohort, located in the north of Sweden, possesses a reduced sensitivity to pain and temperature but little in the way of autonomic disturbances (Einarsdottir et al., 2004; Minde et al., 2004), attributed to a genetic mutation affecting the production of nerve growth factor beta (Einarsdottir et al., 2004). Using the same brushing stimuli employed by Löken et al. (2009), Morrison et al. (2011) found a downward shift in the overall rating profile of patients with HSAN V; they also showed a linear relationship between velocity and pleasantness, in contrast to the inverted U-shape seen in controls: this they interpreted as reflecting a likely reduction in the density of C-tactile afferents. In the current study, we studied more patients (*n* = 15) than Morrison et al. (*n* = 10) and, importantly, studied affective touch at two sites—the forearm and lower leg—and showed disturbances in C-tactile afferent function that suggest that the density of C-tactile afferents is reduced in patients with HSAN III. Why some of our patients presented a flat response profile we cannot explain, but there were no differences in thermal or vibratory thresholds between this group and that of the other HSAN III patients in whom ratings varied as a function of brushing velocity. Evidently, ratings of pleasantness in those patients with a flat response profile cannot be attributed to any stimulus–response functions that relate to peripheral input.

Because our control subjects were studied in a different laboratory, we could not assess thermal and vibration thresholds in these subjects and hence cannot directly compare thresholds in our HSAN III and control subjects. Nevertheless, previous work from our laboratory had shown that thresholds to both hot and cold stimuli applied to the skin are elevated in HSAN III (Hilz and Axelrod, 2000; Hilz et al., 2004). Starting from a baseline temperature of 32 °C, controls could detect warmth with a mean (± SE) temperature difference of 1.5 ± 0.7 °C, whereas for the patients this was 11.0 ± 3.4 °C; corresponding values for detection of cold were 1.7 ± 0.7 °C and 15.5 ± 9.1 °C (Hilz and Axelrod, 2000). In the current study there were no differences in absolute warm and cold thresholds between patients with a flat response profile to brushing and those with a non-flat profile. The same was true for vibration thresholds: while these were almost double those recorded in a large cohort of control subjects (Bloom et al., 1984), as measured at the thumb (median = 5.9 V^2^) and big toe (median = 7.7 V^2^), there were no differences between our two groups of HSAN III patients.

In the patients with HSAN V, sural nerve and skin biopsies revealed a moderate to severe reduction in the number of unmyelinated axons and a moderate reduction of thinly myelinated axons, but no decrease in the number of large diameter cutaneous axons (Minde et al., 2004, 2009). Moreover, unlike the histological evidence of small-fibre loss in skin, these patients have no signs of large-fibre neuropathy; discriminative touch was intact (Löken et al., 2010). Conversely, we have shown slowing of conduction in the sural nerve in HSAN III, suggesting some loss of large-diameter tactile afferents (Macefield et al., 2011) and, as noted above, vibration thresholds were elevated in our patients (Hilz and Axelrod, 2000). Importantly, that the normal rating profile of pleasantness was absent in our HSAN III patients suggests that the density of C-tactile afferents is reduced, which fits with the reported loss of C-fibres in the skin and the elevated thermal thresholds (Hilz and Axelrod, 2000; Hilz et al., 2004).

## 5 Conclusions

We have shown that a specific element of tactile processing, affective touch, is affected in patients with HSAN III, emphasising the diversity of systems affected in individuals with this particular form of hereditary autonomic and sensory neuropathy. In the somatosensory nervous system this well-defined genetic mutation results in a marked reduction in C-fibre density in the skin (cutaneous nociceptor thresholds are greatly elevated and, as shown in the current study, small diameter tactile afferents are also affected), without affecting large-diameter cutaneous afferents. Conversely, the genetic disorder affects *large-diameter* muscle afferents (and results in an apparent agenesis of muscle spindles) yet evidently preserves C-fibre function in muscle (the perception of muscle pain is preserved). It remains to be seen what other systems are affected in HSAN III, but clearly this is one of nature's models that still has surprises in store.

## Figures and Tables

**Table 1:** Thermal and vibratory thresholds for 14 patients with HSAN III, grouped according to their rating profile to stroking (see Fig. 2). COLD = absolute cold threshold, measured from a baseline temperature of 32 °C; WARM = absolute warm threshold, measured from a baseline temperature of 32 °C; VIB = vibration perception thresholds (square of voltage) measured in the right (UR) and left (UL) upper limbs and the right (LR) and left (LL) lower limbs. Median vibration thresholds in control subjects are 5.9 and 7.7 in the upper and lower limbs, respectively. There were no significant differences in thermal or vibratory thresholds between the two groups of HSAN III patients.

Case	sex	age (y)	COLD (°C)	WARM (°C)	VIB UR (V^2^)	VIB UL (V^2^)	VIB LR (V^2^)	VIB LL (V^2^)
*Non-flat rating profile*								
GJ	F	16	28.0	38.4	15	12	16	18
SZ	F	16	27.2	38.2	15	10	10	10
PG	F	19	23.0	37.5	10	10	10	11
JF	M	29	26.5	46.7	15	15	22	28
SF	M	31	27.2	37.5	10	11	28	16
EG	M	38	21.0	35.3	13	8	10	8
JF	F	41	27.0	34.4	12	11	11	11
Mean ± SE		**27.1 ± 3.9**	**25.7 ± 1.0**	**38.3 ± 1.5**	**12.9 ± 0.9**	**11.0 ± 0.8**	**15.3 ± 2.7**	**14.6 ± 2.6**
								
*Flat rating profile*								
RN	F	14	29.2	36.5	9	6	8	12
SL	F	16	7.5	45.1	12	11	11	12
SW	M	22	27.9	35.3	15	15	18	21
JS	F	30	18.0	46.0	19	24	30	34
SS	F	31	30.1	33.3	10	9	10	15
RB	F	34	18.4	43.2	23	27	25	26
BS	M	48	23.7	42.3	11	15	14	11
Mean ± SE		**27.9 ± 4.4**	**22.1 ± 3.1**	**40.2 ± 1.9**	**14.1 ± 2.0**	**15.3 ± 2.9**	**16.6 ± 3.1**	**18.7 ± 3.3**
